# Mobility-Aware Caching and Computation Offloading in 5G Ultra-Dense Cellular Networks

**DOI:** 10.3390/s16070974

**Published:** 2016-06-25

**Authors:** Min Chen, Yixue Hao, Meikang Qiu, Jeungeun Song, Di Wu, Iztok Humar

**Affiliations:** 1 School of Computer Science and Technology, Huazhong University of Science and Technology, Wuhan 430074, China; minchen2012@hust.edu.cn (M.C.); yixuehao@hust.edu.cn (Y.H.); 2 Department of Computer Science, Pace University, New York, NY 10038, USA; mqiu@pace.edu; 3 School of Data and Computer Science, Sun Yat-sen University, Guangzhou 510006, China; wudi27@sysu.edu.cn; 4 Laboratory for Telecommunications, Faculty of Electrical Engineering, University of Ljubljana, Trzaska 25, SI-1000 Ljubljan, Slovenia; iztok.humar@fe.uni-lj.si

**Keywords:** caching, computation offloading, human mobility

## Abstract

Recent trends show that Internet traffic is increasingly dominated by content, which is accompanied by the exponential growth of traffic. To cope with this phenomena, network caching is introduced to utilize the storage capacity of diverse network devices. In this paper, we first summarize four basic caching placement strategies, i.e., local caching, Device-to-Device (D2D) caching, Small cell Base Station (SBS) caching and Macrocell Base Station (MBS) caching. However, studies show that so far, much of the research has ignored the impact of user mobility. Therefore, taking the effect of the user mobility into consideration, we proposes a joint mobility-aware caching and SBS density placement scheme (MS caching). In addition, differences and relationships between caching and computation offloading are discussed. We present a design of a hybrid computation offloading and support it with experimental results, which demonstrate improved performance in terms of energy cost. Finally, we discuss the design of an incentive mechanism by considering network dynamics, differentiated user’s quality of experience (QoE) and the heterogeneity of mobile terminals in terms of caching and computing capabilities.

## 1. Introduction

The ever-growing number of smart phones is producing explosive amounts of traffic in order to support a wide plethora of multimedia services. A recent Cisco report estimates that global mobile traffic will exceed 24.3 exabytes monthly in 2019 [1,2]. However, due to the centralized nature of mobile network architectures, it is challenging to cope with the rapidly growing mobile traffic along with the limited capacity of the backhaul link. In order to overcome this issue, paradigms called “content-centric networking” (CCN), “named data networking” (NDN) and “content delivery networks” (CDN) [3,4] have been proposed to handle content-dominated Internet traffic for the radio access networks (front-haul) and the core networks (back-haul).

Furthermore, alongside the use of diverse network resources [5,6] in terms of communications, caching and computing are becoming the emerging techniques to meet the increasing demand of user QoE (Quality of Experience) in the next generation 5G networks [7,8,9,10,11], especially for the Internet of Things [12,13] and healthcare systems [14]. In this paper, we consider a heterogeneous [15] cellular network, which consists of a Macrocell Base Station (MBS), Small cell Base Stations (SBS) (also called small cell BS; also called as pico, pico- or femto-cells as per the size of the cell) and user terminals. The caching and computing capabilities of SBSs and user terminals will facilitate content sharing and computation offloading.

To illustrate, viral on-line videos are the kind of content that mobile user repeatedly access, which leads us to an assumption that this content could be cached and shared at the edge of the network [16,17,18]. Typically, content caching at the edge of the network can be classified into two categories, i.e., SBS caching (or femto-caching) [19] through femto-cell access points and Device-to-Device (D2D) caching assisted by user terminals [20]. The SBS can be used for content caching, since it is characterized by a high storage capacity and transmission range, and SBS-assisted cache placement has been discussed in previous studies [21]. In addition, by using D2D links, user terminals in the proximity can share cached content without communicating through the MBS in order to reduce communication cost and delay [22]. With the increase of the hardware performance of mobile devices, mobile devices potentially have the storage and computing capacity required for this type of content sharing [23,24]. Various studies discuss cache placement on mobile devices in the D2D networks [25,26].

The problem of caching placement to maximize the probability that the user can access content in a wireless system where both SBSs [19] and user terminals [27,28] have caching capability has been studied. However, most existing studies of caching networks ignore user mobility. Instead, it has been commonly assumed that mobile users are always at a fixed location [29].

In this paper, we investigate the impact of the user mobility on the performance of caching and computation offloading in 5G ultra-dense cellular networks. Then, we propose a joint Mobility-aware and SBS density caching placement scheme (MS-caching), taking into account the impact of user mobility and SBS distribution on the caching placement. Moreover, we addressed the SBS and mobile devices’ computing power, and we summarize four computation offloading modes in 5G ultra-dense cellular networks and propose a hybrid computation offloading strategy. Finally, we discuss an incentive mechanism to encourage content sharing and computation offloading between users with heterogeneous mobile devices. In summary, the contributions of this article include:

- We propose a novel caching placement strategy named MS caching. Then, we discuss the impact of the user mobility and the density of SBS on the content caching.
- We discuss the differences and relationships between caching and computation offloading and present a hybrid computation offloading based on MBS computation offloading, SBS computation offloading and D2D computation offloading.
- Considering the selfishness of mobile users, we suggest an incentive design based on network dynamics, differentiated user’s QoE, and the heterogeneity of user terminals in terms of caching and computing.

The remainder of this article is organized as follows. In Section 2, we present caching in 5G ultra-dense cellular networks followed by the computation offloading in 5G ultra-dense cellular networks in Section 3. Next, an incentive mechanism for both caching and computing is discussed in Section 4. Finally, Section 5 concludes the article.

## 2. Caching in 5G Ultra-Dense Cellular Networks

**Figure 1:**
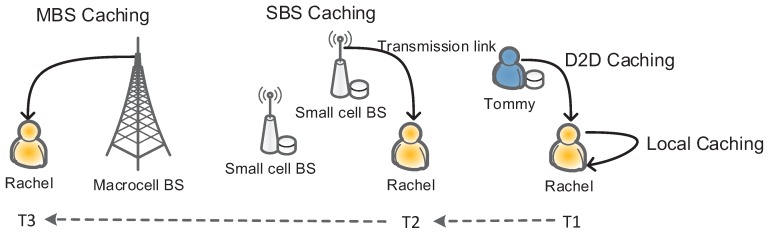
Illustration of the protocol for content access.

In this section, we present the strategy of caching placement by considering the user mobility and SBS density. We assume that each user will randomly request files from one content library containing *l* files $F=\{F_{1},F_{2},\cdots ,F_{l}\}$, and the files are sorted according to popularity, i.e., ranking from the most popular ($F_{1}$) to the least popular ($F_{m}$). Let $|F_{f}|$ denote the size of $F_{f}$. In addition, it is assumed that the popularity of a content requested by a user follows a Zipf distribution with parameter *γ*. i.e.,

(1) $$q_{f}=\frac{f^{-\gamma }}{\sum_{i=1}^{m}f^{-\gamma }},f=1,2,\cdots ,l$$

where *γ* stands for the uneven distribution of popularity in these content. As shown in Figure 1, the user can obtain the requested content mainly via four ways listed as follows:

- *Local caching*: When the user requests content, he or she will firstly examine whether or not such content is cached locally. Once such content is confirmed in the local storage, the user will get access to it without any delay.
- *D2D caching*: If the content requested by the user is not cached locally, the user will seek such content among the devices within the range of D2D communications. If there exists one user caching such content, the content will be transmitted to the target user via D2D communications.
- *SBS caching*: Besides D2D caching, if the required content is cached by one SBS, it will be transmitted to the user by the SBS.
- *MBS caching*: If the content requested by the user cannot be accessed in the aforementioned ways, such a request will be forwarded to the MBS, and the content will be delivered to the user by cellular network connection.

### 2.1. System Model

Given the example in Figure 1, *Rachel* obtains the requested content by one of the means mentioned above when she moves to different locations starting from time T1 to T3. Due to user mobility, the D2D caching is limited by its short distance range, which presents us with the challenge of how to prepare an optimal cache placement strategy, i.e., content caching at the SBS and the user terminal, and how to maximize the chance to access such content.

Now, let us look at the SBS cache placement. Let *R* denote the transmission radius of the SBS; $C_{H}$ denotes the cache capacity of each SBS, i.e., the maximum number of files it can store. Following the model in [30,31], the SBS spatial distribution is in accordance with Poisson Point Processes (PPPs), and its density is *ρ*. In terms of cache placement on the SBS, we can describe it as follows: set $\omega_{i}$ as the probability of caching a file $F_{i}$ in the SBSs. Since the SBSs follow PPPs, the probability of at least one SBS caching the content $F_{i}$ can be calculated as follows:

(2) $$P_{i}^{S}=1-e^{-\rho \omega_{i}\pi R^{2}}$$

Thus, the total probability that a user can get the content from the SBS becomes:

(3) $$P^{S}=\sum_{i=1}^{l}q_{i}P_{i}^{S}$$

If we maximize the probability that the user obtains the content requested under the condition of the storage capacity of SBS, the SBS density-aware caching placement can be obtained as follows:

(4) maximizeωiPSsubject to∑i=1lωi|Fi|≤CH 0≤ωi≤1,i∈{1,⋯,l}

For user terminals, we assume that there are $N_{u}$ mobile devices in this network. Additionally, $D=\{D_{1},D_{2},\cdots ,D_{N_{u}}\}$ represents the set of mobile devices. Communication can only be conducted when the shortest distance between any two mobile devices of users is $R_{D2D}$. Define the inter-contact time $T_{i,j}$ between any two users $D_{i}$ and $D_{j}$ as follows:

(5) $$T_{i,j}=\min\{\left(t-t_{0}\right):||L_{i}^{t}-L_{j}^{t}||<R_{D2D},t>t_{0}\}$$

where $t_{0}$ stands for the moment when the user device $D_{i}$ just the left communication range $R_{D2D}$ of the user device $D_{j}$ for the last time. $L_{i}^{t}$ and $L_{j}^{t}$ stand for the locations when the users $D_{i}$ and $D_{j}$ are in the moment *t*. Following the model in [32], the inter-contact time between any two users $D_{i}$ and $D_{j}$ complies with an exponential distribution with a parameter of $\lambda_{i,j}$, which is named as the contact rate of the mobile device *i* and the mobile device *j*. Let $C_{U}$ denote the cache capacity of each user. Let $x_{j,f}$ denote whether the user *j* caches content $F_{f}$. Let $T_{f}$ denote the deadline to feedback requested content. Thus, within $T_{f}$, the probability that the user *i* obtains the content *f* via D2D can be calculated as follows:

(6) $$P_{i,f}^{M}=1-\left(1-x_{i,f}\right)\exp\left(-\sum_{j\in D\backslash \{D_{i}\}}x_{j,f}T_{f}\lambda_{i,j}\right)$$

Thus, the total probability for the user to get the the content through D2D communication becomes:

(7) $$P^{M}=\frac{1}{N_{u}}\sum_{i=1}^{N_{u}}\sum_{i=1}^{l}q_{f}P_{i,f}^{U}$$

If we maximize the probability that the user obtains the content requested under the condition of the storage capacity of mobile devices, the optimal mobility-aware caching placement can be obtained as follows:

(8) maximizexj,fPMsubject to∑f=1lxj,f|Ff|≤CU xj,f∈{0,1}

Through joint optimization of $P^{M}$ and $P^{S}$, the MS caching strategies can be obtained.

### 2.2. Simulation Results and Discussions

In this subsection, we evaluate the probability that users can get contents via simulation results. We compare MS caching with two different caching strategies: *popular caching* [33] and *random caching* [20].

- *Popular caching*: The popular caching strategies on SBSs and on mobile devices of users are as follows: (1) caching strategy on SBSs: most popular content should be stored on each SBS; (2) caching strategy on mobile devices: most popular content should be cached on each mobile device.
- *Random caching*: The random caching caching strategies on SBSs and on mobile devices of users are as follows: (1) caching strategy on SBSs: content should be stored at random on each SBS; (2) caching strategy on mobile devices: content should be cached at random on each mobile device.

**Figure 2:**
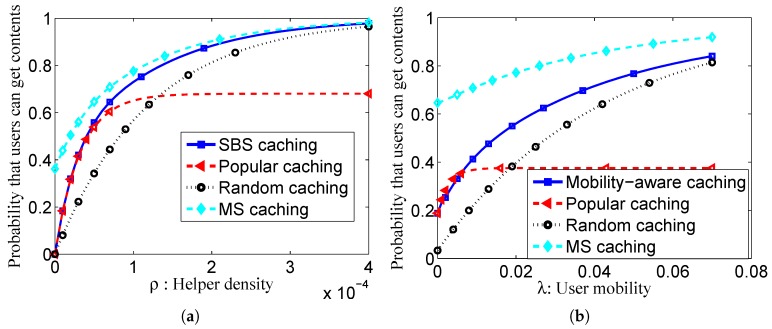
Illustration of the result of caching placement. (**a**) The impact of *ρ* on the probability that users can get content; (**b**) the impact of *λ* on the probability that users can get content.

As for the simulation settings, for simplicity, assume the content size is the same and the value is |F|. The size of content library l=30, and the Zipf distribution parameter γ=0.8. The deadline $T_{f}=60$ s. The density and transmission range of SBSs are $\rho =50/\pi 500^{2}$ and R=50 m, respectively [34]. The system comprises $N_{u}=60$ mobile device, and the contact rate $\lambda_{i,j}$ between user $D_{i}$ and user $D_{j}$ complies with Gamma distribution Γ(4.43,1/1088) [35]. The caching capacity of SBS and the user terminal is $C_{H}=8$ and $C_{U}=2$, respectively. For the optimization problem, we utilize the optimization toolkit CPLEX and CVX to solve it. The result is as follows:

- *SBS density-aware caching placement:* We have provided the relationship between the SBS density and the probability that the user can obtain the requested content. The SBS density-aware caching placement is compared to the popular caching strategy and the random caching strategy, as shown in Figure 2a. When only the SBS is considered, the SBS-assisted cache placement exhibits higher offloading probability than the popular caching and the random caching.
- *Mobility-aware caching placement:* The user’s mobility is closely related to the probability for the user to access the content. The *λ* is the average contact rate of user devices. Similarly, with the analysis of SBS-assisted caching placement, Figure 2b compares the mobility-aware caching with the popular caching and the random caching. As shown in Figure 2b, the mobility-aware cache placement strategy demonstrates better performance than the random caching placement and the popular caching placement.
- *MS caching placement:* If we take into account the user mobility and the SBS density, a more advanced cache strategy named MS caching placement can be designed as demonstrated. In Figure 2a,b, we compare the performance of the proposed MS caching placement with other strategies. Since both the SBS density and the user mobility are considered, the MS caching placement obtains the highest probability that users can obtain the contents.

In Figure 2a, based on the comparison of MS caching, popular caching and random caching, we can obtain the following: (i) as for the density of the SBSs, we cache popular content in a low density region of the SBS, while relatively unpopular content is cached in a high density region to achieve both caching efficiency and a balanced distribution of content; (ii) as for user mobility, the user appears in more locations when his/her mobility is very high, which provides more chances for other users to retrieve the cached content. Thus, a user with high mobility is suggested to cache diverse content, or vice versa, a low mobility user caches popular content.

**Figure 3:**
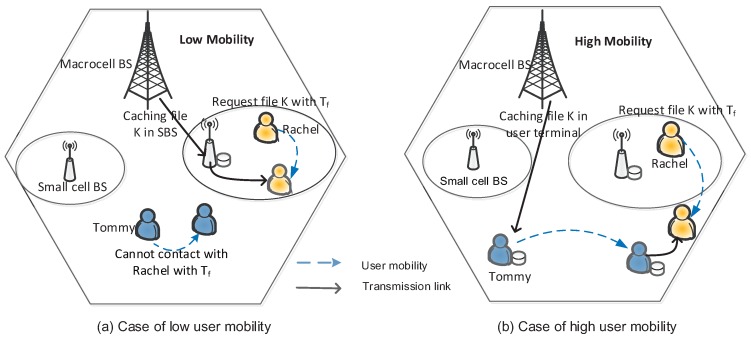
Illustration of the content caching placement. (**a**) Case of low user mobiliity; (**b**) Case of high user mobiliity.

Based on the above discussions, here, we provide an example of content caching when user’s mobility and SBS density are considered. As shown in Figure 3, we differentiate the user mobility in low mobility and high mobility cases. In Figure 3, *Rachel* sends a request to a file K deadline $T_{f}$ since D2D-caching and SBS caching are not available. We assume that the MBS knows the users’ mobility trajectory in the network. If the user mobility is quite low at that moment, the MBS considers the probability for *Rachel* to meet another user (e.g., *Tommy* in Figure 3a carrying the file within $T_{f}$) is low. Then, The MBS transmits the file K to the SBS closest to *Rachel* through a back-haul link, and SBS delivers the cached file to *Rachel*. Figure 3b shows the scenario of high mobility, where the MBS predicts that at least one user will likely come into the vicinity of *Rachel* within $T_{f}$ according to the mobility status in the network. In response to *Rachel*’s request for the file K, she would wait for the D2D opportunity in order to avoid using a more expensive communication channel (e.g., through femtocell caching). After a short while, *Tommy* moves to the D2D communication range and sends the file K to *Rachel*. In the opposite case that *Rachel* still fails to obtain the requested content while the deadline is soon to expire, the MBS will still utilize the traditional SBS caching.

## 3. Computation Offloading in 5G Ultra-Dense Cellular Networks

Mobile cloud computing has been widely studied. Traditional mobile cloud architecture is based on a centralized cloud. For example, in [36] a cloud-assisted drug recommender system is proposed to provide online medical recommendation based on a centralized cloud. However, with the densification of SBSs to cope with ever-growing data traffic, the weakness of this structure is exposed with higher load and more backhaul delay [37]. As one more consequence, communication cost is also increased to offload computing-intensive tasks to the cloud and return the processed result [38]. To solve the problem, previous work also considered the computing capability of the user terminals and the SBSs [32]. In [37,39,40,41], offloading of the computation task to a mobile-edge cloud is investigated with the consideration of delay and energy cost. By comparison, we address the computation offloading issue by means of using the SBS and the user terminal in the 5G ultra-dense cellular networks while the user’s mobility is considered.

### 3.1. Caching vs. Computation Offloading

In this section, we discuss the essential similarities and differences between caching and computation offloading. Content caching is generally provided by the server where the requested content originates from; content is cached during non-peak periods at the MBS, the SBS or the user terminal in order to save the bandwidth in critical time. During “rush hour”, corresponding contents are preferred to deliver to the user via SBS or other user terminal. Furthermore caching and computation offloading are correlative; for example, when the user requests for popular videos, the user terminal or SBS will transmit such content to the user, but the content is found not satisfactory in terms of video quality or the format specially required by the user. The user needs to transcode the original format to the one that satisfies the user. Thus, the task will be offloaded to SBS and/or other user terminals to speedup the computation. Table 1 provides the main differences between caching and computation offloading.

### 3.2. Computation Offloading

In this section, we have summarized the methods of mobile-edge computing offloading assisted by MBS, SBS and the user terminal [37,38,39]. The edge cloud is called the MBS cloud, when it consists of the computing resources deployed in MBS. Similarly, the edge cloud powered by SBS’s computation resources is called the SBS cloud. By comparison, the edge cloud via D2D links is called the mobile cloud.

- *MBS computation offloading* [39]: A user can offload the computation task to an MBS through a cellular network link. In the research area of mobile cloud computing, when the computation is performed in a cloud environment, the results will be fed back to the user from the cloud via the MBS.
- *SBS computation offloading* [37]: The computation task is offloaded to an SBS. After SBS completes the computing, the results will be fed back to the user.
- *D2D computation offloading* [38]: A user terminal can offload the computation task via a D2D link to other mobile devices within the D2D range. Upon the task completion, the results can be transmitted back to the user terminal, if the mobile devices are still within the D2D communication range.

There are some advantages and disadvantages to the the above methods. The MBS computation offloading brings the highest communication cost, but provides the largest coverage [38]. The D2D computation offloading has the lowest cost, but it is difficult to ensure the completion of tasks by taking into account the user mobility. The SBS computation offloading falls somewhere in between. Taking into account the advantages and disadvantages of the above three methods, we have proposed a *hybrid computation offloading*. In the context of the a computation offloading, we name the user terminal that has been assigned the computation task as a computation node and the user terminal processing the computation task as a service node. When the computation node and the service node are within range of the D2D communication, the computation node offloads the computational task to the service node. After a period of time, the service node finishes the assigned task; at this moment, the computation node and service node are possibly out of the range of D2D communication because of user mobility. Thus, in some cases, the service node might be required to cache the computing results for a long time until it again comes into the vicinity of the computation node. On the other hand, if a higher storage capacity and a larger transmission radius of the SBS are available, the computing results can be returned back to the computation node in three manners after the computational task is processed at the service node:

- *D2D computing result feedback:* After the computational task is processed at the service node, the computing results will be returned directly back to the computation node if the service node and the computation node are still within the range of the D2D communication.
- *SBS computing result feedback:* After the computational task is completed at the service node, the service node will offload the computing results onto the SBS if the computation node is out of the range of the D2D communication. Then, the SBS will transmit the results to the user if it is within the communication range with the computation node.
- *MBS computing result feedback:* When the result of the computing task has not been transferred to the user before the deadline, namely when the user and the SBS are still not within the communication range, the SBS will upload the results to the MBS, and then, the final results will be passed back to the user.

**Figure 4:**
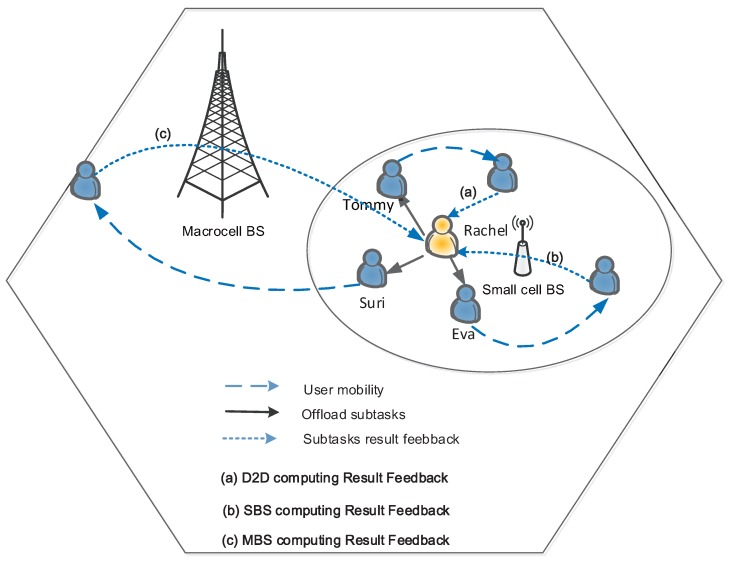
Illustration of the hybrid computation offloading: (**a**) Device-to-Device (D2D) computing result feedback; (**b**) Small cell Base Station (SBS) computing result feedback; (**c**) Macrocell Base Station (MBS) computing result feedback.

As shown in Figure 4, *Rachel* (i.e., the computation node) first divides the computation task into three sub-tasks. Within her D2D communication range, there are three users that can work as service nodes, i.e., *Tommy*, *Eva* and *Suri*. Then, *Rachel* offloads the three sub-tasks to them via D2D links. When a service node (e.g., *Eva*) finishes the computation sub-task, it possibly loses D2D connections with *Rachel* due to the user mobility. Figure 4 gives three modes for the computation result feedback, i.e., the D2D computing result feedback, the SBS computing result feedback and the MBS computing result feedback. After the *Tommy*’ sub-task completion, *Tommy* is still within *Rachel*’s D2D communication range, and the D2D computing result feedback is used. When *Eva*’s sub-task is completed, *Eva* cannot connect with *Rachel* via the D2D link; however, an SBS between *Eva* and *Rachel* is available. Then, the SBS computing result feedback is used. The worst case is the MBS computation result feedback. Given *Suri* as an example, he moves far away, and the cellular network link is the only way to feed back the computation result. Based on the above discussion, we can see that the hybrid computation offloading achieves a flexible tradeoff among D2D computation offloading, SBS computation offloading and MBS computation offloading.

**Figure 5:**
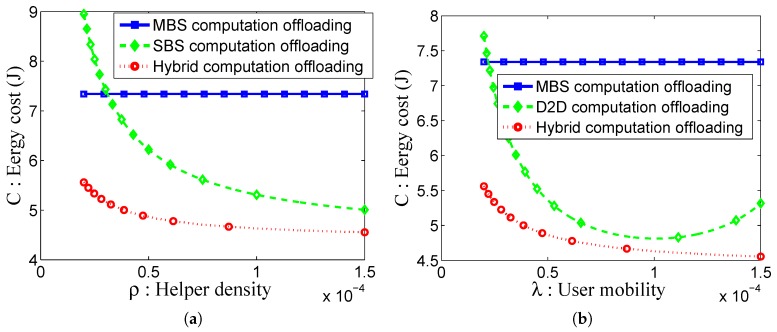
Illustration of the computation offloading energy cost. (**a**) Comparing the energy cost of MBS, SBS and hybrid computation offloading; (**b**) comparing the energy cost of MBS, D2D and hybrid computation offloading.

We consider four kinds of energy consumptions corresponding to four operations during mobile edge computation, i.e., local computing, mobile offloading, edge cloud computing and downloading of computation results from the edge cloud to mobiles. Here, we mainly consider the energy consumption for the mobile terminal. For the four kinds of computation offloading, they have the same local energy consumption. Additionally, edge cloud computing and downloading of computation results do not consume user terminal’s energy. Thus, the major energy consumption of the task is up to mobile offloading. We consider that a user has computation task *Q*, which can be decomposited into *n* sub-tasks. That is: $Q=\sum_{i=1}^{n}x_{i}$. Next, we build up the model to calculate the energy cost of the mobile device. Let $P_{t}^{M}\left(r\right)$, $P_{t}^{S}\left(r\right)$ and $P_{t}^{D}\left(r\right)$ denote the transmission power for the user terminal in terms of the communication via MBS, SBS and D2D, respectively. Let *h* denote the channel gain and $\sigma_{0}^{2}$ denote the variable of complex white Gaussian noise. Then, the channel capacity of the user terminal and MBS can be obtained $C^{M}=B\log\left(1+\frac{P_{t}^{M}\left(r\right)h}{\sigma^{2}}\right)$, where *B* is the channel bandwidth. Likewise, the channel capacity of the user terminal, SBS and D2D can be obtained $C^{S}=B\log\left(1+\frac{P_{t}^{S}\left(r\right)h}{\sigma^{2}}\right)$, $C^{D}=B\log\left(1+\frac{P_{t}^{D}\left(r\right)h}{\sigma^{2}}\right)$. Thus, when obtaining the distance (denoted by r) between the user and MBS, the mobile energy cost for task offloading to the MBS edge cloud is $E_{M}=\sum_{i=1}^{n}\left[\frac{x_{i}}{C^{M}}\left(\frac{1}{\eta }P_{t}^{M}\left(r\right)+P_{c}\right)\right]$, where $P_{c}$ represents the circuit power consumed at the use terminal. Similarly, with the distance between SBS and the user, the mobile energy cost for task offloading to the SBS edge cloud can be calculated as: $E_{S}=\sum_{i=1}^{n}\left[\frac{x_{i}}{C^{S}}\left(\frac{1}{\eta }P_{t}^{S}\left(r\right)+P_{c}\right)\right]$. With higher small cell density, the user has more chance to offload the task onto a small cell with a closer distance and less energy cost. For the case of D2D, if the distance between two adjacent users is known, the D2D energy cost for the task offloading is $E_{D}=\sum_{i=1}^{n}\left[\frac{x_{i}}{C^{D}}\left(\frac{1}{\eta }P_{t}^{D}\left(r\right)+P_{c}\right)\right]$. With the increasing of user mobility, the user terminal with a shorter distance will be found for task offloading, which decreases the energy cost. In order to produce optimal performance, the location of task offloading is strategically selected in the hybrid cloud, which exhibits the lowest energy cost. According to [37,39], we set the total task amount Q=10 Mbytes and n=10. Let B=1 MHz, $\sigma^{2}=10^{-9}$ W, $h=10^{-5}$. Set the maximum transmit power of the user terminal $P_{max}=1$ W, and the circuit power $P_{c}=115.9$ mW. The result as shown in Figure 5.

In Figure 5, we evaluate the performance of the MBS computation offloading, the SBS computation offloading, the D2D computation offloading and the hybrid computation offloading in terms of communication cost. With the increase of SBS density, the cost of the SBS computation offloading and the hybrid computation offloading decrease since higher SBS density facilitates the computation result offloading to the SBS, as shown in Figure 5a. Figure 5b shows the impact of the user mobility on the energy cost. With the increase of the user mobility, both the D2D computation offloading and the hybrid computation offloading exhibit lower energy cost. This is because the probability of the D2D connections increases. The performance of the D2D computation offloading achieves optimization when *λ* is equal to 0.00011. However, the energy cost increases again when the mobility is too high. This is because the contact time of the D2D connection is too short, which easily causes the failure of the computation result feedback. In comparison, the hybrid computing offloading combines the advantages of the other three computation offloading schemes and produces the lowest energy cost.

## 4. Incentive Design for Caching and Computation Offloading

As already mentioned, the main target of caching and computation offloading in 5G ultra-dense cellular networks is to reduce traffic load and encourages the D2D communications among users. However, the intrinsic selfish feature of user terminals constitutes the biggest obstacle for content caching and computation offloading in practical situations. For example, most users intend to store their favorite files, which at the same time might be also cached by many other users. This fact could result in replicated caching and insufficient use of the accumulative storage space of the network nodes cumulatively. As for computing, most users like to count on others to help them to execute the computation tasks while being reluctant to share computing capacity with others.

In order to solve the problem, this paper designs an incentive mechanism based on the following three kinds of heterogeneities: (1) the heterogeneity of the user devices, namely each user terminal’s storage and computing capabilities are different, which makes some users willing to cache contents and earn incentives through content sharing, while other users prefer to provide computing service to others, and the earned incentive can be used to request cached contents; (2) the heterogeneity of user requirements in terms of user’ QoE, namely each user’s demand for computing and caching and his/her preference for content are different; (3) the heterogeneity of network conditions, namely the user mobility within the region and the density of the SBSs are different.

Similar to the incentive mechanism for crowdsourcing, more incentives lead to a higher user’s QoE. There are two main methods to earn the incentive: cache content and computing tasks for others. Moreover, the two can be transformed into each other; for example, user *Bob*’s mobile phone has large storage capacity to cache more popular content, but its computing capacity is relatively weak; whereas, user *Suri*’s mobile phone has great computing capacity, but weaker storage capacity. When both are within the range of the D2D communication, user *Bob* may offload some content as requested by user *Suri* and then get some incentive when the content is sent to *Suri*. Thereby, user *Bob* may offload computational tasks to be processed onto user *Suri*, and user *Suri* can get some incentive, which can pay for the “debt” for getting the content. Therefore, an incentive balance of content is achieved and replaced by computation. We introduce an incentive mechanism to encourage various users with heterogeneous mobile devices to exchange favors of content sharing and computation offloading.

Specifically, we can divide this incentive into two levels:

- *Caching incentive*: When the user B obtain content from the user A, the user B needs to pay an incentive (e.g., virtual money), this incentive includes the cost of D2D communication between B and A, the cost of storing content at the expense of the content value from the perspective of the user A. Meanwhile, the user A can get these incentive. From the above, we can see that the popularity of the content of the caching incentive, downloading times of users and caching time are all related to these three aspects.
- *Computing incentive*: When the user A offloads computing tasks and transfers them to the user B and then the user B helps the user A to proceed with the calculation, the user A will pay the user B an incentive for the communication cost and the computing cost. At the same time, the user B will obtain these incentives. The costs are relatively high due to the fact that the result of computation is equivalent to the content, whose popularity is zero.

## 5. Conclusions

With the increasing capabilities of mobile terminals in terms of storage and computing, caching of popular content on wireless devices enables content sharing through the D2D links. Even though various works focus on caching placement in the 5G ultra-dense cellular networks, it is still a challenging issue to jointly consider caching and computing by using the advantage of the user mobility. In this article, we proposed an MS caching placement with the use of the SBS and the user terminal while taking the effect of user mobility and SBS density into consideration for content caching. Then, we designed a hybrid computation offloading scheme to achieve flexible tradeoffs among the MBS computation offloading, the SBS computation offloading and the D2D computation offloading. Finally, we discussed an incentive design in terms of caching and computing by considering the hardware heterogeneity of the mobile devices, various user’s requirements on QoE and the heterogeneity status of the network.

In future work, we will consider the social relationship of the user terminal in the D2D communication. It can be concluded from the users’ social relationship that those with social connections tend to have the same request for content; for example, one region may be divided into different groups, such as an industrial group, a tourism group, a residential group, etc. Different contents will be cached in different regions, and in the same region, the interchange of content may be better achieved.

## Figures and Tables

**Table 1:** Caching vs. computation offloading.

Caching	Computation Offloading
No feedback, one-way cache and fetch.	Need the feedback of the computation result.
The popularity of the cached content is typically high.	The popularity of cached computation result can be understood as 0, since it usually only serves one particular user.
The size of shared storage is relatively large.	The shared space to store the computation result is relatively small.
